# Metformin and Vascular Diseases: A Focused Review on Smooth Muscle Cell Function

**DOI:** 10.3389/fphar.2020.00635

**Published:** 2020-05-08

**Authors:** Mingying Deng, Dan Su, Suowen Xu, Peter J. Little, Xiaojun Feng, Liqin Tang, Aizong Shen

**Affiliations:** ^1^The First Affiliated Hospital of USTC, Division of Life Sciences and Medicine, University of Science and Technology of China, Hefei, China; ^2^School of Pharmacy, The University of Queensland, Woolloongabba, QLD, Australia; ^3^Department of Pharmacy, Xinhua College of Sun Yat-sen University, Guangzhou, China

**Keywords:** cardiovascular diseases, metformin, vascular smooth muscle cells, atherosclerosis, pulmonary hypertension

## Abstract

Metformin has been used in diabetes for more than 60 years and has excellent safety in the therapy of human type 2 diabetes (T2D). There is growing evidence that the beneficial health effects of metformin are beyond its ability to improve glucose metabolism. Metformin not only reduces the incidence of cardiovascular diseases (CVD) in T2D patients, but also reduces the burden of atherosclerosis (AS) in pre-diabetes patients. Vascular smooth muscle cells (VSMCs) function is an important factor in determining the characteristics of the entire arterial vessel. Its excessive proliferation contributes to the etiology of several types of CVD, including AS, restenosis, and pulmonary hypertension. Current studies show that metformin has a beneficial effect on VSMCs function. Therefore, this review provides a timely overview of the role and molecular mechanisms by which metformin acts through VSMCs to protect CVD.

## Introduction

Metformin is a biguanide that is available from extracts of folk medicinal plant *Galega officinalis* leaves (Palmer and Strippoli, 2018; Prattichizzo et al., 2018). This medicinal plant has been used for hundreds of years because of its ability to inhibit bacteria, viruses, and malaria as well as antipyretic and analgesic (Prattichizzo et al., 2018; Flory and Lipska, 2019; Soukas et al., 2019). Metformin has been widely used to treat diabetes since the 1950s and was widely used in the United States in 1995, which greatly promoted the study of application and mechanism of metformin (Nafisa et al., 2018; Soukas et al., 2019). Metformin is the most common treatment for type 2 diabetes (T2D) in the world (Rena and Lang, 2018). This is mainly because of its high clinical value and low cost in controlling blood glucose (Maruthur et al., 2016).

In addition to treating T2D, metformin is also used to treat pre-diabetes, polycystic ovary syndrome (PCOS), gestational diabetes, and to treat or prevent of pre-eclampsia, as well as to prevent weight gain or even weight loss in diabetic patients (Lord et al., 2003; Syngelaki et al., 2016; Greenhill, 2018). A growing number of studies have shown that the beneficial effects of metformin on health exceed the improvement in blood glucose levels (Bannister et al., 2014; Campbell et al., 2017). Diabetes patients treated with metformin showed survival benefit even compared with non-diabetic controls (Bannister et al., 2014; Campbell et al., 2017). Human observations further support the role of metformin in preventing aging and cancer (Castillo-Quan and Blackwell, 2016; Campbell et al., 2017; Pryor et al., 2019).

In patients with T2D, metformin monotherapy has lower morbidity and mortality associated with cardiovascular disease compared to sulfonylurea monotherapy, and metformin combined with sulfonylurea therapy is associated with a reduced risk of fatal cardiovascular events (Johnson et al., 2005). Adolescents with T1D exhibit insulin resistance and impaired vascular health (including increasing ascending and descending aorta pulse wave velocity and maximal [WSS_MAX_] and time-averaged [WSS_TA_] wall shear stress, and decreasing ascending aorta and descending aorta relative area change) (Bjornstad et al., 2018). Metformin can improve body mass index (BMI), body weight, fat mass, insulin dose, and aortic and carotid health in T1DM adolescents, thereby improving insulin resistance (Bjornstad et al., 2018). Metformin is expected to play a cardioprotective role in T1D (Bjornstad et al., 2018). Sardu *et al*. assessed the effects of metformin treatment on coronary endothelial function (coronary diameter of the left anterior descending coronary artery [LAD]) and major adverse cardiac events (MACE) (including heart death, myocardial infarction, and heart failure) in patients with pre-diabetes with stable angina pectoris and non-obstructive coronary stenosis (NOCS) (Sardu et al., 2019). Metformin treatment can reduce coronary endothelial dysfunction and reduce the risk of MACE in patients with pre-diabetes (Sardu et al., 2019).

Metformin is a first-line treatment for lowering glucose, and human and animal studies have shown that metformin can inhibit gluconeogenesis by blocking mitochondrial redox shuttles and fructose-1,6-bisphosphatase-1 (FBP1) in the liver (Hunter et al., 2018; Flory and Lipska, 2019). Metformin is also an insulin sensitizer and may play a protective role in the intestinal lumen through a variety of mechanisms (Flory and Lipska, 2019; Horakova et al., 2019). Metformin can also reduce low density lipoprotein cholesterol (LDL-C) and total cholesterol (TC) levels (Xu et al., 2015; Weng et al., 2020); inhibit inflammatory responses (such as inhibiting the activation of nuclear factor kappa B [NF-κB] and interleukin-1β [IL-1β]) (Isoda et al., 2006; Deng et al., 2018); improve vascular endothelial function (activate 5'-adenosine monophosphate-activated protein kinase [AMPK], increase nitric oxide [NO] synthesis) (Nafisa et al., 2018; Sardu et al., 2019); inhibit cardiac remodeling (such as inhibiting cardiomyocytes apoptosis and cardiac fibrosis) (Sasaki et al., 2009; Chen R. et al., 2018). These suggest that metformin has a cardiovascular protective effect, but a comprehensive understanding of the mechanism of action of metformin is still lacking (Rena et al., 2017; Flory and Lipska, 2019).

Vascular smooth muscle cells (VSMCs) are a class of cells that are highly malleable and multifunctional (Touyz et al., 2018; Basatemur et al., 2019). The healthy VSMC is located in the middle of the artery wall and has a fusiform shape that expresses contractile proteins (such as smooth muscle myosin heavy chain [SMMHC] and α-smooth muscle actin [αSMA] as well as secretes ECM macromolecules, including elastin, collagen, and proteoglycans (Owens et al., 2004; Touyz et al., 2018). VSMCs play an important role in compliance and elastic rebound when adapting to changes in hemodynamic conditions (Touyz et al., 2018; Basatemur et al., 2019; Xu et al., 2019). VSMCs function is a key factor determining the characteristics of the entire arterial vascular. Physiological VSMCs are at rest and exhibits low levels growth (Coll-Bonfill et al., 2016; Basatemur et al., 2019). Overgrowth of VSMCs contributes to the onset of several types of CVD, including AS, restenosis, and pulmonary hypertension (PAH) (Singh et al., 2002; Rudijanto, 2007; Tajsic and Morrell, 2011).

In this article, we focus on the effects of metformin on vascular function by regulating VSMCs function, including vasoconstriction/PAH, intimal thickening, vascular calcification, and inflammation.

## Effect of Metformin on Vascular Function/Diseases

### Vascular Tone/Activity

Dilation blood vessels are a commonly used method in the treatment of clinical hypertension (Mughal and O'Rourke, 2018). Vascular contraction and relaxation are regulated by a variety of factors, among which vasoactive substances such as endothelin synthesized and released by vascular endothelial cells can cause vascular smooth muscle contraction by increasing intracellular free calcium ([Ca^2+^]i) levels in vascular smooth muscle cells (Ali and Khalil, 2015). Vasodilators, such as NO and prostacyclin (Prostacyclin I_2_, PGI_2_), released by vascular endothelial cells, which have opposite functions to endothelin, cause vasodilation by reducing the [Ca^2+^]i concentration in vascular smooth muscle cells (Ali and Khalil, 2015; Vanhoutte et al., 2017). [Ca^2+^]i in vascular smooth muscle cells is mainly derived from extracellular calcium entering through the cell membrane calcium channels and intracellular calcium released from the endoplasmic reticulum of vascular smooth muscle cells (Guo et al., 2018; Touyz et al., 2018; Manoury et al., 2020). In addition to calcium channels, there are also K^+^ channels on the vascular smooth muscle cell membrane, which affects the vascular smooth muscle cell membrane potential to regulate the vasomotor function of the vessels (Manoury et al., 2020). When external stimulation causes the K^+^ channel on the cell membrane to open, the cell membrane potential appears hyperpolarized, which can inhibit the voltage-dependent calcium channel opening on the membrane, reduce extracellular calcium influx, and promote vasodilation (Zhu et al., 2018; Manoury et al., 2020). Conversely, the K^+^ channel is closed, the cell membrane potential is depolarized, and the Ca^2+^ entering the cell through the voltage-dependent calcium channel on the membrane increases, resulting in vasoconstriction (Zhu et al., 2018; Manoury et al., 2020).

Diabetic and hyperlipidemia rats exhibit high reactivity in vasoconstriction at an early stage (Li et al., 2015). Hyperglycemia and hyperlipidemia directly affect the contractile function of VSMCs, suggesting that early damage of VSMCs during metabolic disease may play a key role in vascular dysfunction (Li et al., 2015). A large proportion of people with diabetes have high blood pressure (Bohm et al., 2019; Saydah et al., 2019). Methylglyoxal is a highly reactive dicarbonyl produced in the metabolism of fructose and glucose, which is also the main precursor for the formation of glycation end products (AGEs) (Schalkwijk and Stehouwer, 2020). Methylglyoxal levels are elevated in the plasma of diabetic patients and hypertensive rats (Dhar et al., 2013). In VSMCs, methylglyoxal stimulation can up-regulate protein expression of α1D receptor and AT1 receptor (Dhar et al., 2013). Metformin, a methylglyoxal scavenger and AGEs inhibitor, attenuates the effects of methylglyoxal (Dhar et al., 2013).

In VSMCs, insulin and insulin-like growth factor I (IGF-1) inhibited agonist-stimulated contraction and increased [Ca^2+^]i (Dominguez et al., 1996). In hypertensive rats, metformin can promote the antihypertensive effect of peripheral insulin (Dominguez et al., 1996). In cultured rat aorta VSMCs, metformin increased basal tyrosine kinase (TK) activity, glucose transport, and decreased thrombin-induced [Ca^2+^]i elevation (Dominguez et al., 1996). These insulin/IGF-like effects may be helpful in understanding certain vascular protection effects of metformin (Dominguez et al., 1996). Similarly, in cultured VSMCs of rat thoracic aorta, metformin treatment significantly reduced angiotensin II (ANG II) or platelet-derived growth factor (PDGF)-stimulated [Ca^2+^]i, suggesting metformin exerts its vasodilating effect by inhibiting agonist-induced [Ca^2+^]i (Sharma and Bhalla, 1995). In the rat tail artery, metformin acute relaxation of phenylephrine (PE)-induced contraction is accompanied by repolarization of the arterial VSMCs membrane (Peuler et al., 1999). The acute relaxation of metformin on rat tail artery smooth muscle may depend on the transmembrane K gradient (metformin fails to relax the extracellular K-induced contraction) and mediated at least by 4-aminopyridine (4AP)-sensitive voltage-dependent K^+^-channel activation in the arterial VSMCs membrane (Peuler et al., 1999).

The effect of metformin on relaxing blood vessels is affected by the type of inducer. For example, in rabbit arteries, metformin activates AMPK without inhibiting cyclopropazine (CPA)-induced arterial contraction and does not inhibit [Ca^2+^]i (Huang et al., 2017). In addition, in the single cell isolated from the mesenteric resistance artery of guinea pigs, the thiazolidinedione derivatives (troglitazone and pioglitazone) inhibited the contraction induced by 77 mM K^+^, and its potency was similar to that of inhibiting Ca^2+^ current. Metformin and bezafibrate have no significant effect on Ca^2+^ current or high K^+^ induced contraction (Nakamura et al., 1998). In VSMCs and pancreatic beta cells, phenformin but not metformin inhibits many K_ATP_ variants (Aziz et al., 2010).

These studies suggest that metformin relaxes vascular smooth muscle mechanisms: enhances insulin sensitivity, inhibits methylglyoxal activation of the renin angiotensin system, and inhibits multiple inducers (including PDGF or ANG II)-stimulated [Ca^2+^]i rise, as well as inhibits PE-induced [K^+^]i rise. It is precisely because of the antihypertensive effect of metformin by inhibiting [Ca^2+^]i and/or [K^+^]i, so metformin and bezafibrate have no significant effect on Ca^2+^ current or high K^+^ induced contraction (Nakamura et al., 1998).

In addition, in SHR tail artery tissue (rich in sympathetic nerve endings [SNE]), the vasodilation effect of metformin on smooth muscle is attenuated by the presence of SNE (Lee and Peuler, 2001). Further studies have shown that metformin has an indirect sympathomimetic effect, which affects its vasodilation (Lee and Peuler, 2001). Moreover, the indirect sympathomimetic effect can be amplified by a monoamine oxidase inhibitor (such as promethazine) and blocked by a NE-carrier inhibitor (such as desipramine), so that metformin has beneficial vasodilatation in diabetic patients with hypertension may be affected by commonly used antidepressants similar to promethazine and desipramine (Lee and Peuler, 2001).

### Vascular Calcification

Vascular calcification (deposition of hydroxyapatite minerals in the arterial wall) is closely related to the increased risk of stroke, heart disease, and atherosclerotic plaque rupture (Nicoll and Henein, 2014; Chow and Rabkin, 2015). Calcification occurs in the intimal and medial layers of the arteries, both driven primarily by VSMCs (Schinke and Karsenty, 2000; Chow and Rabkin, 2015; Durham et al., 2018).

Atherosclerosis-associated calcification is closely related to plaque rupture (Durham et al., 2018; Mori et al., 2018). Genetic defects in AMPKα1 but not AMPKα2 promote atherosclerosis calcification and Runt-related transcription factor 2 (Runx2) expression (Cai et al., 2016). AMPK activation increases Runx2 instability by promoting small ubiquitin-like modification of Runx2 (SUMOylation) (Cai et al., 2016). In contrast, long-term administration of metformin significantly reduced atherosclerotic calcification and Runx2 expression in ApoE^(-/-)^ mice, but less in ApoE^(-/-)^/AMPKα1^(-/-)^ mice (Cai et al., 2016). At the same time, AMPKα1 deficiency in VSMCs increases Runx2 expression and promotes atherosclerotic calcification *in vivo* (Cai et al., 2016).

Proliferation and migration of VSMCs are significant in the development of AS and plaque rupture (Durham et al., 2018; Harman and Jorgensen, 2019). In primary human aortic myocytes (HASMCs), metformin-induced AMPK activation inhibits proliferation and migration of HASMC by up-regulating tumor suppressor protein p53 (p53) and interferon-inducible protein 16 (IFI16) (Hao et al., 2018). SiRNA-mediated knockdown of p53 and IFI16 attenuated AMPK activation and reversed the inhibition of metformin (Hao et al., 2018). These findings suggest that metformin may have therapeutic potential in AS (Hao et al., 2018).

In female rat aortic smooth muscle cells (RASMCs), metformin attenuated β-glycerophosphate (β-GP)-stimulated alkaline phosphatase activity and calcium deposition, while reducing osteoblast-like genes (Runx2 and bone morphogenetic protein-2) expression and increasing specific markers of muscle cells (alpha-actin) expression (Cao et al., 2013). Mechanistic studies indicated that metformin increased phosphorylation levels of AMPK and endothelial nitric oxide synthase (eNOS), and nitric oxide (NO) production (Cao et al., 2013). When pharmacological methods are used to inhibit AMPK or eNOS, NO production is reduced and metformin-mediated vascular protection against β-GP-stimulated calcium deposition is eliminated (Cao et al., 2013). This evidence indicated that metformin blocks vascular calcification through the AMPK/eNOS/NO signal pathway and may have therapeutic potential for vascular calcification in T2D complications (Cao et al., 2013).

Maintaining mitochondrial homeostasis may be a potential protective factor for VSMCs to resist osteoblast-like phenotypic transitions (Jia et al., 2018; Zhu et al., 2019). In VSMCs, supplementation with metformin restores β-GP-mediated mitochondrial biogenesis in VSMCs, such as increased mitochondrial DNA copy number, up-regulated mitochondrial membrane potential (MMP), and mitochondrial biosynthesis gene expression (Ma et al., 2019). Metformin inhibits pyruvate dehydrogenase kinase 4 (PDK4)/oxidative stress-mediated apoptotic pathway through enhanced mitochondrial biogenesis, thereby attenuating β-GP-induced phenotype conversion of VSMCs into an osteogenic phenotype (Ma et al., 2019).

### Intimal Thickening

Diabetes is a very important risk factor for CVD, which is closely related to VSMCs hyperproliferation and intimal thickening (Torella et al., 2018; Ji et al., 2019). Two types of microRNAs (miR-221/222) that promote intimal thickening were found in the inner mammary artery (IMA) segment of 37 subjects who underwent coronary artery bypass grafting (Coleman et al., 2013). These patients included non-diabetic patients (ND), diabetic patients who took metformin (DMet+), and diabetic patients who did not take metformin (DMet−) (Coleman et al., 2013). Compared to the ND and DMet+ groups, the DMet− group showed an up-regulation of miR-221/222 and a down-regulation of their downstream target p27 mRNA (Coleman et al., 2013). The level of miR-221/222 was inversely related to the dose of metformin. The proliferation rate of VSMCs isolated from the IMM of the DMet− group was faster than that of the ND and DMet+ groups (Coleman et al., 2013).

Proliferation of VSMCs caused by vascular injury plays an important role in the formation of vascular lesions (Durham et al., 2018; Zhang C. et al., 2018). Peroxisome proliferator-activated receptor γ coactivator-1 (PGC-1) is a very important regulator of many biological processes such as energy metabolism (Islam et al., 2018; Lee et al., 2019). PGC-1β expression was decreased in the carotid artery of rats with balloon injury (Guo et al., 2013). Overexpression of PGC-1β *in vivo* significantly inhibits neointimal formation and significantly reduces proliferation of VSMCs (Guo et al., 2013). Furthermore, metformin can inhibit the proliferation of VSMCs by up-regulating the expression of PGC-1β (Guo et al., 2013). In a rat model of insulin resistance formed by high fructose diet for 4 weeks, the rat carotid artery was damaged by balloon catheter. Metformin treatment significantly attenuates neointimal hyperplasia by inhibiting VSMCs proliferation, migration, and inflammation, and improving insulin signaling pathways (Lu et al., 2013). In addition, hyaluronic acid (HA) is an extracellular matrix glycosaminoglycan that is involved in a variety of biological processes such as cell proliferation, inflammation, and vascular thickening (Vigetti et al., 2011; Chen L. H. et al., 2018). Metformin greatly reduces HA synthesis by activating AMPK. The reduction in HA reduces the ability of human aortic smooth muscle cells (HASMCs) to proliferate, migrate, and recruit immune cells, thereby reducing the atherogenic HASMCs phenotype (Vigetti et al., 2011).

The above studies indicate that metformin has therapeutic potential neointimal thickening after arterial injury. However, some studies do not support this conclusion. For example, in a rat carotid balloon injury model, metformin enhanced insulin sensitivity but had no significant effect on neointimal thickness in normal or high-fat diet-fed rats (Guo et al., 2017). Similarly, metformin (10 mmol/L) activated AMPK and inhibited VSMCs proliferation only in undamaged blood vessels, while metformin (2 mmol/L) had no effect (Guo et al., 2017). These results show that although metformin increases systemic insulin sensitivity, it does not reduce intimal growth after arterial injury in rats (Guo et al., 2017). Abnormal arterial SMCs proliferation is associated with AS and intravascular restenosis, which is common in diabetic patients (Peuler et al., 1996). One study compared the direct effects of glibenclamide, pioglitazone, thiazolidinediones, and metformin on the proliferation of cultured arterial SMCs (Peuler et al., 1996). The results indicate that pioglitazone may be more useful than glibenclamide and metformin (Peuler et al., 1996).

### Inflammatory Response

Cytokine and chemokine-stimulated VSMCs accelerate the inflammatory response and migrate to the damaged endothelium during AS progression (Das et al., 2018). Phosphatase and tensin homolog (PTEN) play a negative regulatory role in inflammation (Sisti et al., 2018; Tang et al., 2018). In VSMCs, metformin inhibits tumor necrosis factor-α (TNF-α)-stimulated inflammatory response (inhibition of nuclear factor [NF]-κB) activation, and expression of inducible nitric oxide synthase (iNOS) and cyclooxygenase (COX)-2 *via* AMPK-induced PTEN expression (Kim and Choi, 2012).

Increased vascular cell oxidative stress is associated with the pathogenesis of AS (Forstermann et al., 2017; Tawakol and Jaffer, 2018). Reactive oxygen species (ROS) stimulate vascular inflammation through the pro-inflammatory cytokine/NF-κB pathway (Kim et al., 2007; Zhong et al., 2019). Peroxisome proliferator activated receptor γ coactivator-1-alpha (PGC-1α) plays a very important role in the regulation of intracellular ROS levels (Garcia-Quintans et al., 2014; Garcia-Quintans et al., 2016). TNF-α plays a major pro-inflammatory role in vascular inflammation, and it increases NADPH oxidase activity and mitochondrial ROS level (Kim et al., 2007; Blum and Adawi, 2019). PGC-1α overexpression in endothelial cells (HAECs) and HASMCs inhibits TNF-α-induced mitochondrial ROS production, NADPH oxidase activity, NF-κB activity, and monocyte chemotactic protein-1 (MCP-1) and VCAM-1 expression (Kim et al., 2007). Expression of PGC-1α in HASMCs and HAECs can be enhanced by AMPK activators, such as metformin, rosiglitazone, and alpha-lipoic acid (Kim et al., 2007). Therefore, metformin may prevent the development of AS by stimulating PGC-1α expression in the vasculature (Kim et al., 2007).

In addition, metformin dose-dependently inhibited the production of pro-inflammatory cytokines IL-6 and IL-8 in IL-1β-induced VSMCs, macrophages (Mphis), and endothelial cells (ECs) (Isoda et al., 2006). Metformin exerts a direct vascular anti-inflammatory effect by blocking the phosphatidylinositol 3-kinase (PI3K)/protein kinase B (Akt) signaling pathway and subsequently inhibiting NF-κB activity (Isoda et al., 2006). This direct anti-inflammatory effect of metformin may partly explain the significant reduction in clinical cardiovascular events by metformin, which is not entirely due to its hypoglycemic effect (Isoda et al., 2006).

In addition to its anti-inflammatory effects in VSMCs, metformin inhibits lipopolysaccharide (LPS)-induced NF-κB activation, expression and secretion of cytokines and chemokines (including TNF-α, IL-1α, T cell activation gene-3 [TCA-3], macrophage colony-stimulating factor [M-CSF]) in mouse colonic SMCs (CSMCs) (Al-Dwairi et al., 2018). The anti-inflammatory effect of metformin in CSMCs suggests that it can be used as adjunctive therapy for patients with inflammatory bowel disease (a recurrent inflammation of the intestine and the structure and function of intestinal SMCs are significantly affected) (Al-Dwairi et al., 2018).

### Atherosclerosis (AS)

AS is the formation of plaques containing lipids, cells, debris, and scar tissue in the arterial intima (Zhang L. et al., 2018; Feng et al., 2019a; Feng et al., 2019b). Endothelial dysfunction, smooth muscle cell proliferation and migration, monocyte adhesion and macrophage inflammation, cholesterol accumulation to form foam cells, and platelet aggregation are all important links in the pathogenesis of AS (Feng et al., 2019b; Feng et al., 2020). It causes major pathological processes of stroke, angina pectoris, myocardial infarction, and heart failure and is now the leading cause of death worldwide (Zhang L. et al., 2018; Basatemur et al., 2019; Feng et al., 2019a; Feng et al., 2019b). The excessive proliferation and migration of VSMCs are important factors that cause neointimal hyperplasia and lumen stenosis after vascular injury (Feng et al., 2019b).

In *Intimal Thickening*, *Inflammatory Response*, and *Atherosclerosis (AS)*, we have summarized that metformin can improve the intimal thickening, vascular calcification, and inflammation by affecting the function of VSMCs, all of which are important pathological features of AS. Recently, Jenkins *et al*. have summarized randomized clinical trial reports on the effects of metformin on the markers of atherosclerotic vascular disease, including carotid intima-media thickness (CC-IMT), vascular reactivity and calcification in patients with type 1 diabetes (T1D) and T2D (Jenkins et al., 2018). These studies generally indicate that metformin has a protective effect on vascular disease in young and adults with T1D, and in adults with pre-diabetes and T2D (Jenkins et al., 2018).

The effect of metformin on AS, from the perspective of VSMCs, includes: reduction of intimal thickening, reduction of inflammation and oxidative stress, prevention of atherosclerosis calcification. In addition, metformin can also improve endothelial dysfunction (including inhibition of oxidative stress and inflammatory response, inhibition of ECs senescence and apoptosis, and improvement of ECs NO production and endothelium-dependent vasodilation) (Nafisa et al., 2018).

### Pulmonary Arterial Hypertension (PAH)

Pulmonary arterial hypertension (PAH) is a debilitating disease associated with increased pulmonary arterial pressure, decreased lung function and exercise capacity, and progressive right heart failure (Pullamsetti et al., 2014; Yuan et al., 2019). Endothelial dysfunction, vasoconstriction, pulmonary vascular remodeling caused by smooth muscle cell hyperplasia and hypertrophy, muscleization of the anterior capillaries, and distal vascular loss are key pathophysiological processes of PAH (Tuder et al., 2013; Sundd and Kuebler, 2019). PAH causes increased pulmonary vascular resistance and increased right ventricular afterload, and promotes right ventricular failure, which is the main cause of death of patients with PAH (Thenappan et al., 2018). Current treatments, including calcium channel blockers, anticoagulants, endothelin receptor antagonists, type 5 phosphodiesterase inhibitors, and prostacyclin analogs, have limited efficacy (Thenappan et al., 2018). Vascular occlusion caused by excessive proliferation of VSMCs is an important feature of pulmonary hypertension (Uhrin et al., 2018). In addition to dilating blood vessels, it is also necessary to inhibit the inflammatory response and reverse pulmonary vascular remodeling (Uhrin et al., 2018).

Pulmonary artery vascular smooth muscle (PAVSM) cell proliferation is an important pathological process of PAH vascular remodeling (Krymskaya et al., 2011; Kudryashova et al., 2016). Metformin has relaxed blood vessels and anti-proliferative properties (Agard et al., 2009). In hypoxic and monocrotaline-induced rat PAH models, metformin is resistant to vascular remodeling (including improved endothelial function, vasodilation, and anti-proliferative effects) and resistance to PAH (Agard et al., 2009). Similarly, in the sugen 5416/hypoxia-induced PAH rat model, metformin inhibited right ventricular systolic pressure and hypertrophy, as well as reduced pulmonary vascular remodeling (Dean et al., 2016). This may be related to inhibition of aromatase activity, estrogen synthesis and cell proliferation in PAVSM cells (Dean et al., 2016). In the hypoxic experimental PAH rat model, metformin (100 mg/kg/day) treatment for 3 weeks also inhibited hypoxia-induced pulmonary vascular remodeling, collagen deposition, and PAVSM cells proliferation by activating AMPK and inhibiting autophagy (Liu et al., 2019).

Up-regulation of mammalian target of rapamycin (mTOR) activity and activation of mammalian target of rapamycin complex 1 (mTORC1) and mTORC2 are important for the proliferation of PAVSM cells stimulated by chronic hypoxia *in vitro* and *in vivo* (Krymskaya et al., 2011). In PAVSM cells, metformin activates AMPK, inhibits mTOR activity, and reverses the up-regulation of S-phase kinase-associated protein 2 (Skp2) and down-regulation cyclin-dependent kinase inhibitor 1B (p27), thereby inhibiting PDGF or endothelin-1 (ET-1) induced proliferation (Wu et al., 2014; Song et al., 2016). These studies suggest that metformin has potential value in the prevention and treatment of PAH by negatively regulating pulmonary vascular remodeling (Wu et al., 2014; Song et al., 2016). Metformin is currently used to treat patients with diabetes, so it should be easier to assess its use as a treatment for PAH in diabetic patients (Agard et al., 2009).

## Molecular Targets of Metformin

**Figure 1:**
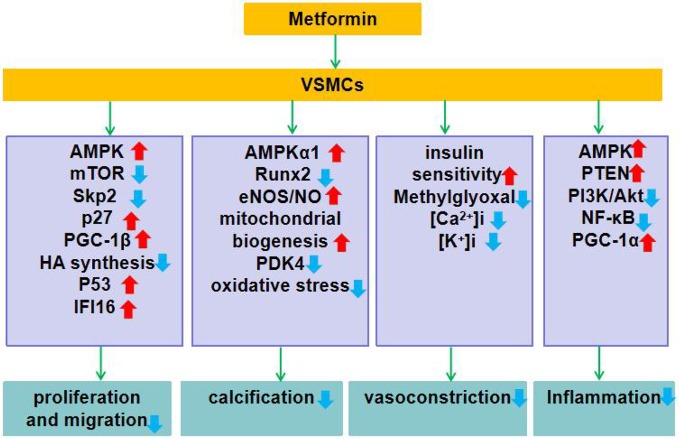
Role of metformin in VSMCs dysfunction. Metformin exerts its function of improving the function of VSMCs by regulating the expression and function of genes or proteins closely related to VSMCs proliferation and migration, calcification, contraction, and inflammation. ↑indicates increase or activation, and ↓indicates decrease or suppression. Metformin inhibits VSMCs proliferation: 1. Activates AMPK, inhibits mTOR, down-regulates Skp2 while up-regulates p27. 2. Up-regulates expression of PGC-1β. 3. Activates AMPK, reduces HA synthesis. 4. Activates AMPK, up-regulates P53 and IFI16. Metformin inhibits vascular calcification: 1. Activates AMPKα1 and inhibits Runx2 expression. 2. Activates the AMPK-eNOS-NO pathway. 3. Enhances mitochondrial biogenesis and inhibits PDK4/oxidative stress-mediated apoptotic pathway. Metformin inhibits smooth muscle contraction: 1. Enhances insulin sensitivity and inhibits methylglyoxal activation of renin angiotensin system. 2. Inhibits the rise of [Ca^2+^]i and [K^+^]i in VSMCs. Metformin inhibits inflammation: 1. Activates AMPK and up-regulates PTEN expression. 2. Inhibits PI3K-Akt and NF-κB activation. 3. Up-regulates the expression of PGC-1α.

In general, metformin reduces vascular remodeling (by inhibiting VSMCs proliferation, migration, calcification, and inflammation), and dilates blood vessels (by enhancing insulin sensitivity and inhibiting the rise of intracellular [Ca^2+^]i and [K^+^]i). Although the exact molecular mechanism of metformin is still not well defined, many secondary molecular targets have been identified so far. Here, we summarize the molecular targets of the action of metformin on VSMCs (**Figure 1**).

## Conclusion and Perspectives

In this article, we focus on the role of metformin in CVD, including AS and PAH, and clarify that metformin plays a beneficial role in the above CVD by improving the function of VSMCs. In general, metformin inhibits proliferation, calcification, and inflammation of VSMCs. However, more research is needed to support the beneficial effects of metformin on VSMCs. In addition, we summarized the mechanism of action of metformin on VSMCs (**Figure 1**).

Metformin has been used in diabetes for more than 60 years (Flory and Lipska, 2019). Newer drugs, including glucagon-like peptide 1 (GLP-1) receptor agonists and sodium glucose cotransporter 2 (SGLT-2) inhibitors, also show cardiovascular benefits (Association A D, 2019). However, the safety of long-term application of these new drugs remains to be confirmed by further studies, such as SGLT-2 inhibitors may cause diabetic ketoacidosis and amputation, and GLP-1 agonists may cause acute pancreatitis (Association A D, 2019). In contrast, metformin's clinical experience and safety data are much better (UK Prospective Diabetes Study [UKPDS] Group, 1998; Knowler et al., 2002). In fact, the common gastrointestinal side effects of metformin are usually transient, and the only serious adverse reaction is lactic acidosis, which occurs nine times per 100,000 person-years (Stang et al., 1999; Triggle and Ding, 2017).

Given the safety, effectiveness, and significant cost advantages of metformin, it remains the first-line therapy for most T2D patients (Flory and Lipska, 2019). In addition, it is clinically possible to control the dose of metformin, use sustained-release and delayed-release preparations, and manage patients to alleviate the common side effects of metformin (Flory and Lipska, 2019). In the future, relying on modern pharmacological methods, human and animal studies will further promote the rational use of metformin and reveal the mechanism of action of metformin.

## Author Contributions

LT and AS directed the writing and were responsible for the overall guidance. MD and DS searched and organized the literature, and wrote the manuscript. PL and SX proofreaded and revised the manuscript. MD and XF wrote and revised the manuscript.

## Conflict of Interest

The authors declare that the research was conducted in the absence of any commercial or financial relationships that could be construed as a potential conflict of interest.

## Abbreviations

AMPK, 5'-adenosine monophosphate-activated protein kinase; Akt, protein kinase B; [Ca^2+^]i, intracellular free calcium; eNOS, endothelial nitric oxide synthase; HA, hyaluronic acid; IFI16, interferon-inducible protein 16; [K^+^]i, intracellular free Potassium; mTOR, the mammalian target of rapamycin; NF-κB, nuclear factor kappa B; NO, nitric oxide; p27, p53, tumor suppressor protein p53; cyclin-dependent kinase inhibitor 1B; PDK4, pyruvate dehydrogenase kinase 4; PGC-1α, Peroxisome proliferator-activated receptor γ coactivator-1α; PGC-1β, Peroxisome proliferator-activated receptor γ coactivator-1β; PI3K, phosphatidylinositol 3-kinase; PTEN, Phosphatase and tensin homolog; Runx2, Runt-related transcription factor 2; Skp2, S-phase kinase-associated protein 2.
